# Adaptation and validation of the Hungarian version of Thyroid-Related Patient-Reported Outcome-39 (ThyPro-39) questionnaire: testing factor structure, known-group validity with the comparison of quality of life in Hashimoto’s thyroiditis and Graves’ disease

**DOI:** 10.1186/s41687-023-00606-7

**Published:** 2023-06-27

**Authors:** Adrien Rigó, Katalin Malkov, Alexandra Szabó, Virág Katalin Bognár, Róbert Urbán

**Affiliations:** 1 Institute of Psychology, Eötvös Loránd University, Izabella utca 46, Budapest, H-1064 Hungary; 2 Institute of Behavioural Sciences, Faculty of Medicine, Semmelweis University, Budapest, Hungary

**Keywords:** Autoimmune thyroid disease, Hashimoto’s disease, Graves’ disease, Health-related quality of life, ThyPro-39

## Abstract

**Background:**

Living with autoimmune thyroid disease is a longstanding challenge and can seriously affect the quality of life. We aimed to adapt and validate the Hungarian version of the Thyroid-Related Patient-Reported Outcome-39 (ThyPro-39) questionnaire, test its factor structure, and compare two frequent autoimmune thyroid diseases, Hashimoto’s thyroiditis, and Graves’ disease. We tested the factor structure of ThyPro-39 with a series of confirmatory factor analyses (CFAs). To examine the validity of ThyPro-39 and to compare the quality of life of the two groups — Hashimoto’s thyroiditis (N = 240), Graves’ disease (N = 51) — CFA with covariates were used.

**Results:**

Our results supported a bifactor model with psychosocial and somatic symptoms as general factors, and 12 symptom-specific factors. Based on the analysis of omega hierarchical indices ranging between 0.22 and 0.66, the specific scales also carry information besides the composite scores and should be used when a more detailed analysis is required. In the multivariate analysis, perceived stress was significantly associated with the general psychosocial factor (β = 0.80), symptom factors (β = 0.34), anxiety (β = 0.43), depressivity (β = 0.37), and emotional susceptibility (β = 0.38) specific factors. Graves’ patients reported more eye symptoms (d = 0.45) and cosmetic complaints (d = 0.40), while Hashimoto patients had more cognitive problems (d = 0.36) and more severe hypothyroid symptoms (d = 0.35). These group differences confirm the known-group validity of the questionnaire.

**Conclusions:**

The validity of the Hungarian version of ThyPRO-39 is supported. We recommend using two composite scores of psychosocial and somatic symptoms and the specific symptoms scores to measure the quality of life in clinical practice and research.

## Introduction

Autoimmune thyroid disorders (AITDs) occur in about 0.3–1.5/1,000 persons/year, with female dominance. AITDs are influenced by genetic, environmental, and epigenetic factors [1–3]. The frequency of AITDs has shown an increasing tendency in different parts of the world in recent decades [4–6]. Rapidly changing environmental factors can be mainly responsible for this increase [3].

The two most frequent types of AITD are Hashimoto’s thyroiditis (HT) and Graves’ disease (GD). Although the underlying autoimmune mechanisms are similar in the two disorders, the targets of the autoantibodies are different, and as a result, the symptoms are also different [1, 7]. HT causes hypothyroidism in about 20–30% of patients [2]. In GD the unregulated thyroid hyperfunctioning is characteristic of the disease, which may also be accompanied by orbital and pretibial extrathyroidal manifestations [8]. Because HT and GD are longstanding diseases with fluctuating disease activity and changing symptoms, they require close medical monitoring and control to avoid severe side effects [2, 6].

Living with autoimmune thyroid disease can seriously affect the health-related quality of life (HRQoL) [9, 10], including disturbing physical symptoms of hypo- or hyperthyroidism, mood-related problems, sexual dysfunctions, neurocognitive disturbances, and cosmetic complaints. Consequently, daily and social functioning are frequently impaired [9–12]. Since symptoms and their effects on well-being and comorbid difficulties vary depending on the inflammatory activity, hormone level changes, and tissue damage, it is necessary to regularly monitor the burden experienced by patients. Age and time since diagnosis can also be important factors in determining symptoms and quality of life. A long-lasting autoimmune process can cause progressive changes in the thyroid gland and brain leading to increased amounts of some types of symptoms. Age and time since diagnosis can also affect coping and adaptation to the disease [9, 13].

Measuring HRQoL in thyroid patients with relatively easy-to-apply self-reported scales can bring many benefits. Disease-specific health-related quality of life (HRQoL) questionnaires offer a quick and broad impression for physicians about the primary and most stressful symptoms, can help to follow some signs of disease activity and the effects of the treatment on symptoms and well-being, and can provide information about the need for mental health care [10, 14–17].

Specifically, in autoimmune thyroid diseases, monitoring HRQoL can be extremely important because hormone levels — the medical parameter checked routinely — do not always provide accurate information about the effects of the illness on well-being. HRQoL seems more related to underlying autoimmune activity or comorbid symptoms and disorders than hormone levels or changes [18–20]. Based on systematic review articles, the Thyroid-Related Patient-Reported Outcome (ThyPro) is recommended for assessing HRQoL in patients with different benign thyroid diseases and dysfunctions [10, 21].

Although ThyPro shows good psychometric characteristics, content, and structural and cross-cultural validity [10, 21, 22], the need for an abbreviated version has arisen [23]. The short form of the ThyPro (ThyPro-39) is based on the original questionnaire and was constructed with item response theory and validation studies. The exploration of the psychometric characteristics of the abbreviated version has just recently begun [10, 24, 25].

Our study aimed to adapt and validate the Hungarian version of the ThyPro-39 questionnaire, test its factor structure [23], and explore its construct validity (including known-group validity) in the two most frequent autoimmune thyroid diseases: HT and GD. Perceived stress is expected to be associated with the general factors and psychosocial specific factors only, and not expected to be associated with the specific symptom factors.

## Materials and methods

### Procedure and participants

The study procedures were approved by the Scientific and Research Ethics Committee of the Hungarian Medical Research Council (SE TUKEB 256/2021) and were completed in accordance with the Declaration of Helsinki as revised in 2013. We recruited our participants from disease-specific groups on social media sites. Informed participant consent was obtained.

The study included data from 291 participants. Of these, 82.5% (N = 240) had an HT diagnosis, and the proportion of GD patients was 17.5% (N = 51). 96.2% (N = 280) claimed to be female. The mean age was 45.1 years (SD = 12.0; range: 22–78). The detailed sociodemographic data are shown in Table 1.

**Table 1:** Demographic and disease-specific characteristics of the recruited sample (N = 291)

Place of living	
Capital	91 (31.3%)
Other cities	147 (50.5%)
Village	50 (17.2%)
Other	3 (1%)
Education	
Primary school	4 (1.4%)
Vocational or high school	111 (38.1%)
College or university BA	101 (34.7%)
College or university MA PhD	70 (24.1%) 5 (1.7%)
Marital status	
Single	32 (11%)
In relationship	236 (81.1%)
Divorced	20 (6.9%)
Widow	1 (0.3%)
Other	2 (0.7%)
Sex	
Male	10 (3.4%)
Female	280 (96.2%)
Other	1 (0.3%)
Type of disease	
Hashimoto’s thyroiditis	240 (82.5%)
Graves’ disease	51 (17.5%)
Time since the diagnosis, years Mean (SD)	6.74 (6.89)
History of thyroid removal surgery	22 (7.6%)
Comorbidities with other chronic conditions	109 (37.5%)
Lifetime psychiatric diagnosis	59 (20.3%)

*Note.* Missing data is not included in the table and the summation of percentages

### Measures

#### Thyroid-Related Patient-Reported Outcome-39

HRQoL was measured with ThyPro-39 questionnaire (24). The participants responded on a five-point Likert scale (0 = no symptoms; 4 = severe symptoms). The questionnaire contains 12 scales and an individual item measuring the overall HRQoL impact. A scale with an additional composite summary score was also created by the original authors [23]. The composite scale is based on the 22 items from the tiredness, cognitive problems, anxiety, depressivity, emotional susceptibility, impaired social, and daily life scales plus the overall HRQoL impact item. The use of the composite score can be useful when simplicity of reporting combined with small measurement intervals and high precision is the goal. In the previous research, this version showed good test–retest reliability and adequate responsiveness to clinical change [23]. Before the start of the research, the questionnaire was officially translated into Hungarian based on the instructions and supervision of the original authors (two independent Hungarian translations - a common translation based on the two Hungarian versions - back translation into English - evaluation by the original authors - reconciled Hungarian version - cognitive debriefing - final Hungarian version).

#### Perceived Stress Scale-10

The Hungarian version of the Perceived Stress Scale-10 (PSS-10) was used to measure the stress level [26, 27]. A higher score indicates more frequent stressful situations; the total score is a global indicator of perceived stress.

#### Mental Health Continuum Short Form

The Mental Health Continuum Short Form (MHCS-SF) is a scale measuring emotional well-being, consisting of 14 items [28, 29]. Overall, the questionnaire describes the subjective well-being of the respondent globally (MHCS-Total), while its three scales (Hedonic — Emotional, Eudaimonic — Social, Eudaimonic — Psychological) summarise each well-being area separately.

### Statistical analysis

Data were analysed with SPSS 26 (IBM, 2017) and Mplus 8.5. To test the measurement models, we used a series of confirmatory factor analyses (CFAs) with maximum likelihood estimation robust to nonnormality (MLR). The models were interpreted following standard goodness-of-fit indices (30): the Comparative Fit Index (CFI; ≥ 0.95 excellent, ≥ 0.90 adequate), the Tucker–Lewis index (TLI; ≥ 0.95 excellent, ≥ 0.90 adequate), and the Root Mean Square Error of Approximation (RMSEA; ≤ 0.06 excellent, ≤ 0.08 adequate). Whenever applicable, χ2 difference test and sample-size adjusted Bayesian Criterion (ssaBIC) value were used to compare alternative measurement models. The χ2 scaled difference test is used in the context of CFA to compare nested models when the data may not meet the assumptions of normality or independence. A straightforward manual calculation for the chi-squared difference test for the MLR estimator is available on the Mplus website. The sample-size adjusted Bayesian Information Criterion (BIC) is a statistical measure used in model selection to compare the goodness-of-fit of competing models. It balances the goodness-of-fit and model complexity by penalizing models that have more parameters, and provides a quantitative measure for selecting the best-fitting model among a set of candidates. A lower sample-size adjusted BIC (ssaBIC) value indicates a better trade-off between goodness-of-fit and model complexity, with lower values indicating better-fitting models among the set of candidates being compared. The practical application of the questionnaire raised the possibility of using a composite score. To test the reliability and validity of the composite score, we tested a bifactor model similar to others [23]. The bifactor model simultaneously represents a general severity factor and the problems or symptoms represented by the specific factors. The usual specification of a bifactor model requires that the specific factors do not correlate with each other or the general factor [30]. The advantage of bifactor modelling is that it provides an opportunity to quantify the appropriateness of a composite score. Therefore, we applied the percent of common variance attributable to the general factor using an explained common variance index (ECV) [31, 32]. We also used omega and omega hierarchical indices to measure how precisely a self-reported symptom scale score assesses the combination of general and specific constructs and a certain target construct [33]. The interpretation of ECV, omega, and omega hierarchical indices is moderated by the percentage of uncontaminated correlations (PUCs). In the case of a high PUC value (> 0.90), the indices can be interpreted directly.

The construct validity of ThyPro-39 is being tested using a CFA with covariates model. The CFA with covariates model allows for estimating the effects of grouping variables, such as diagnosis, or other continuous variables on the latent variables, which can be used to investigate known-group validity while controlling for other covariates. Additionally, perceived stress, age, and sex are included as additional variables to provide further evidence of the construct validity of the measurement.

## Results

### Descriptive statistics

The descriptive characteristics are shown in Table 2. Most scales show acceptable or good internal consistency. Table 2 also contains the comparison of the HT and GD groups on different variables. The correlations among the main variables can be found in Table 3.

**Table 2:** Descriptive statistics of the scales

Scale	Cronbach-α	Items	Mean (SD) Total sample	Mean (SD) Hashimoto (n = 240)	Mean (SD) Graves (n = 51)	t-value	Cohen’s d
*Thyroid-Related Patient-Reported Outcome-39*							
Goitre symptoms	0.83	3	3.03 (3.13)	3.15 (3.19)	2.45 (2.79)	1.46	0.22
Hyperthyroid symptoms	0.66	4	5.57 (3.66)	5.43 (3.60)	6.21 (3.90)	1.39	0.21
Hypothyroid symptoms	0.62	4	7.44 (3.78)	7.67 (3.73)	6.35 (3.87)	2.28*	0.35
Eye symptoms	0.79	3	4.34 (3.51)	4.07 (3.37)	5.62 (3.87)	2.91*	0.45
Tiredness	0.85	3	7.78 (3.13)	7.78 (3.11)	7.78 (3.25)	0.002	0.00
Cognitive problems	0.88	3	4.42 (3.18)	4.62 (3.25)	3.49 (2.63)	2.66**	0.36
Anxiety	0.90	3	5.75 (3.43)	5.76 (3.42)	5.70 (3.51)	0.11	0.02
Depressivity	0.78	3	5.16 (2.33)	5.10 (2.31)	5.41 (2.47)	-0.85	0.13
Emotional susceptibility	0.71	3	6.32 (2.93)	6.24 (2.93)	6.72 (2.93)	-1.08	0.16
Impaired social life	0.79	3	3.01 (2.91)	2.96 (2.89)	3.23 (3.04)	-0.61	0.01
Impaired daily life	0.85	3	4.05 (3.10)	4.08 (3.16)	3.94 (2.84)	0.29	0.04
Cosmetic complaints	0.82	3	4.16 (3.72)	3.90 (3.59)	5.39 (4.09)	-2.62**	0.40
Psychosocial symptoms scale (Composite scale)	0.94	22	38.59 (16.74)	38.57 (16.98)	38.67 (15.69)	-0.03	0.01
Somatic symptom scale	0.85	17	28.17 (12.12)	28.10 (12.27)	28.43 (11.53)	-0.18	0.03
*Perceived Stress* (PSS10)	0.89	10	19.07 (5.93)	18.98 (5.86)	19.51 (6.29)	− 0.058	0.09
*Mental Health Continuum Scale* (MHCS)							
Emotional	0.89	3	11.08 (3.52)	11.22 (3.35)	10.40 (4.19)	1.30	0.23
Social	0.76	5	15.29 (5.39)	15.41 (5.19)	14.72 (6.24)	0.73	0.13
Psychological	0.87	6	24.48 (6.43)	24.62 (6.31)	23.82 (6.98)	0.79	0.12
MHCS_Total	0.91	14	50.84 (13.19)	51.25 (12.71)	48.94 (15.24)	1.00	0.17
				%	%	χ^2^	
Thyroid surgery Other chronic somatic disease				4.6 40.5%	21.6% 21.6%	17.36** 6.66**	

*Note*. *p < .05; **p < .01.; PSS10: Perceived Stress Scale-10; MHCS: Mental Health Continuum Scale; SD: standard deviation; Items: number of items

**Table 3:** Correlations among the main well-being variables

	ThyPro39_ Psychosocial symptoms	ThyPro39_ Somatic symptoms	PSS-10	MHCS_ Total	MHCS_ Emotional	MHCS_ Social
ThyPro39_Psychosocial symptoms	**-**					
ThyPro39_Somatic symptoms	0.64**	**-**				
PSS-10	0.77**	0.38**	-			
MHCS_Total	− 0.50**	− 0.22**	− 0.60**	-		
MHCS_Emotional	− 0.57**	− 0.31**	− 0.60**	0.79**	-	
MHCS_Social	− 0.32**	− 0.12*	− 0.41**	0.84**	0.51**	-
MHCS_Psychological	− 0.45**	− 0.17**	− 0.58**	0.90**	0.64**	0.61**

*Note*. *p < .05; **p < .01.; Table shows Spearman correlations; ThyPro39: Thyroid-Related Patient-Reported Outcome-39; PSS-10: Perceived Stress Scale-10; MHCS: Mental Health Continuum Scale

### Measurement models: confirmatory factor analyses

The measurement model was tested by a series of CFAs. The model fit indices are presented in Table 4. The model with 12 correlated first-order factors (Model 1) showed a good fit. As an alternative model, we examined the fit of a bifactor model that included an overall general factor in addition to the 12 specific factors (Model 2b). Although this model also showed an acceptable fit to the data, the chi-squared difference statistic indicated a better fit for the simpler model (Model 1).

**Table 4:** Model fit coefficients of the CFA models

Models	χ^2^(df) [Scaling correction]	RMSEA [90% CI]	CFI	TLI	SRMR	ssaBIC
Full scale:						
Model 1: Twelve-factor model	879.1 (599) [1.0440]	0.040 [0.034-0.046]	0.944	0.934	0.052	31,118
Model 2a: Bifactor model: one general and twelve specific factors#	1009.1 (628) [1.0536]	0.046 [0.040-0.051]	0.924	0.915	0.069	31,190
Model 2b Bifactor model: one general and twelve specific factors# *with one non-specific item*	1109.1 (665) [1.0520]	0.048 [0.043-0.053]	0.916	0.906	0.068	31,918
Model 3a: Bifactor model: two general and twelve specific factors##	923.8 (629) [1.0556]	0.040 [0.034-0.046]	0.941	0.934	0.072	31,100
Model 3b: Bifactor model: two general and twelve specific factors## *with one non-specific item*	1038.5 (666) [1.0536]	0.044 [0.039-0.049]	0.929	0.921	0.074	31,843
Psychosocial factor:						
Model 4: Seven-factor model	336.6 (168) [1.0488]	0.059 [0.050-0.068]	0.948	0.935	0.047	15,716
Model 5a: Bifactor model: one general factor and seven specific factors	327.8 (169) [1.0746]	0.057 [0.048-0.066]	0.951	0.939	0.055	15,713
Model 5b: Bifactor model: one general factor and seven specific factors *with one non-specific item*	377.1 (189) [1.0720]	0.058 [0.050-0.067]	0.945	0.933	0.055	16,470
Somatic symptom factor:						
Model 6: 5-factor model	182.6 (109) [1.0835]	0.048 [0.036-0.060]	0.947	0.934	0.056	15,497
Model 7: Bifactor model: one general factor and five specific factors	129.3 (103) [1.0957]	0.030 [0.007-0.045]	0.981	0.975	0.042	15,455

*Note*: RMSEA = root mean square error of approximation. CFI = comparative fit index. TLI = Tucker and Lewis index of fit. SRMR = standardized root mean squared residual. #: The residual variance of TQ7c item is constrained in zero to avoid a negative residual. ##: The residual variances of TQ7c and TQ1c items are constrained in zero to avoid negative residuals. The negative residuals may indicate identification problems with these models

Comparison of Models 4 and 5a resulted Δχ2 = 0.14, Δdf = 1, p = .706, it indicates that there is no statistical difference in the degree of fits, however sample-size adjusted BIC value is lower in the bifactor model favoring the bifactor measurement model

Comparison of Models 6 and 7 resulted Δχ2 = 64.3, Δdf = 6, p = < 0.001 in favor for Model 7, and in addition the sample-size adjusted BIC value is also lower in this latter model

Noting that the factors in the measurement model can be grouped into two major categories, namely somatic symptoms and psychosocial factors, we examined the fit of the model containing two general factors — Somatic symptoms and Psychosocial symptoms — in addition to the 12 factors. The fit of the model is slightly weaker than that of Model 1 based on the chi-squared difference statistic, but it has a better fit using information criteria (e.g., ssaBIC).

Since an item in the questionnaire (item 12) is not linked to a specific factor, we also tested measurement models in which this general item only loads on its corresponding general factor (Model 2b and Model 3b). A comparison of the fit of these models with the base model (Model 1) and the previous bifactor models (Model 2a and Model 3a) is not possible because they do not have the same item set.

We also tested the two parts of the questionnaire separately. Thus, on the one hand, we tested a model with seven first-order correlates that represented only Psychosocial symptoms (Model 4). We also tested a bifactor version (Model 5a), which now exhibited a slightly better fit. On the other hand, we tested the model with the five primary somatic symptom factors and its bifactor version. Again, the bifactor model showed a better fit to the data. Thus, the two bifactor models will be presented in further analyses.

The standardised factor loadings of the bifactor model with somatic symptoms and the main indices associated with the bifactor analysis are presented in Table 5. All items loaded significantly on the general factor. For the specific factors, all items loaded significantly except two items. The general factor explained 41% of the common variance (ECV). This value supports the presence of a general factor. The omega indices ranged from 0.70 to 0.90. The omega hierarchical can indicate the added meaning of specific factors over the general factor. These indices were high for the Goitre symptoms, Cosmetic complaints, and Eye symptoms factors and relatively low for Hyperthyroid and Hypothyroid symptoms.

**Table 5:** Bifactor model of somatic symptoms

Items (abbreviated)	General factor	Goitre symptoms	Hyperthyroid symptoms	Hypothyroid symptoms	Eye symptoms	Cosmetic Complaints
Fullness in neck	**0.470**	**0.439**				
Pressure in throat	**0.480**	**0.877**				
Discomfort swallowing	**0.548**	**0.571**				
Trembling hands	**0.356**		**0.354**			
Tendency to sweet	**0.467**		**0.272**			
Palpitation	**0.445**		**0.750**			
Upset stomach	**0.557**		0.108			
Cold sensitivity	**0.295**			**0.182**		
Swollen hands/feet	**0.522**			0.067		
Dry skin	**0.443**			**0.810**		
Itchy skin	**0.491**			**0.451**		
Dryness, grittiness in eyes	**0.502**				**0.499**	
Impaired vision	**0.510**				**0.646**	
Light sensitivity	**0.467**				**0.547**	
Changed appearance	**0.479**					**0.693**
Others gazing you	**0.323**					**0.644**
Change in worn clothes	**0.457**					**0.649**
*ECV*	0.41	0.14	0.09	0.10	0.11	0.15
*Omega*	0.90	0.86	0.70	0.70	0.80	0.83
*Omega hierarchical*	0.73	**0.53**	0.28	**0.30**	**0.45**	**0.59**
*Relative omega*	0,80	0,61	0,40	0,43	0,57	0,71

Note: Percent of uncontaminated correlations (PUC) = 0.846. ECV = explained common variance

The standardised factor loadings of the model measuring psychosocial symptoms and the main indices associated with the bifactor analysis are presented in Table 6. All items loaded significantly on the general factor. The items also loaded significantly on their corresponding specific factors except for one item. The general factor explained 57% of the common variance (ECV). This value supports the presence of a strong general factor. The omega reliability indices ranged from 0.46 to 0.93. The high value of the omega hierarchical index was obtained by the Cognitive problems and Emotional susceptibility factors. The factors Anxiety, Depressivity, and Impaired social life still showed an acceptable value, while Tiredness and Impaired daily life did not reach our predefined criterion level.

**Table 6:** Bifactor model of psychosocial symptoms

Items (abbreviated)	General factor	Tiredness	Cognitive problems	Anxiety	Depressivity	Emotional suspectibility	Impaired social life	Impaired daily life
Been tired	**0.598**	**0.546**						
Difficulty getting motivated	**0.675**	**0.550**						
Energetic	**-0.606**	**-0.453**						
Problems remembering	**0.341**		**0.688**					
Slow or unclear thinking	**0.421**		**0.775**					
Difficulty concentrating	**0.527**		**0.729**					
Afraid or anxious	**0.626**			**0.523**				
Felt tension	**0.658**			**0.609**				
Uneasy	**0.694**			**0.585**				
Sad	**0.729**				**0.333**			
Unhappy	**0.653**				**0.692**			
Self-confident	**-0.555**				-0.127			
Easily stressed	**0.627**					**0.725**		
Mood swings	**0.634**					**0.331**		
Felt in control	**-0.709**					**0.184**		
Difficult being with other people	**0.587**						**0.533**	
A burden to other people	**0.599**						**0.447**	
Conflicts with other people	**0.533**						**0.447**	
Difficulty managing daily life	**0.720**							**0.413**
Difficulty participating in life	**0.686**							**0.643**
Everything takes longer	**0.599**							**0.358**
ECV	0.57	0.06	0.11	0.07	0.04	0.05	0.05	0.05
Omega	0.93	0.46	0.89	0.91	0.57	0.64	0.79	0.87
Omega hierarchical	0.79	0.22	**0.66**	**0.39**	**0.31**	**0.54**	**0.32**	0.29
Relative omega	0.85	0.48	0.74	0.43	0.54	0.84	0.41	0.33

Note: Percent of uncontaminated correlations (PUC) = 0.900. ECV = explained common variance

### Construct validity of general and specific factors: a confirmatory factor analysis with covariate analysis

To test the construct validity of the general and specific factors, we conducted a CFA with covariate analysis using age, gender, diagnosis, and perceived stress to explain the 12 primary and two general factors. The standardised coefficients are presented in Table 7. For the analysis, we applied the traditional p < .05 significance level; however, in the more detailed interpretations, we will only consider those covariates that reach the p < .01 level.

**Table 7:** CFA with covariates model

	Age	Sex	Diagnosis	Perceived Stress	*R* ^*2*^
Psychosocial symptoms factor	0.12*	0.10*	0.11*	0.80***	63.8%
Somatic symptoms factor	0.20**	-0.01	-0.15*	0.34***	14.5%
Tiredness	-0.20	-0.07	-0.20	-0.35	18.2%
Cognitive problems	-0.27***	-0.09	-0.27***	-0.34*	24.0%
Anxiety	-0.25***	-0.02	-0.11	0.43***	30.3%
Depressivity	-0.07	0.02	-0.07	0.37**	15.7%
Emotional suspectibility	-0.12*	-0.02	-0.08	0.38***	18.3%
Impaired social life	-0.13	-0.04	-0.12	0.01	3.4%
Imapired daily life	-0.01	-0.13	-0.24*	-0.32	17.4%
Goitre symptoms	-0.10	-0.01	-0.03	-0.07	1.3%
Hyperthyroid symptoms	-0.01	0.06	0.32**	0.17	12.8%
Hypothyroid symptoms	0.19*	0.20*	-0.01	-0.02	9.4%
Eye symptoms	0.30***	0.01	0.34***	0.03	20.7%
Cosmetic complaints	-0.09	0.11	0.31**	0.09	11.3%

Note: N = 286. Standardized regression coefficients. Sex is coded: 1 = Male; 2 = Female. Diagnosis is coded: 1 = Hashimoto; 2 = Graves). *:p < .05; **:p < .01; ***:p < .001. To keep Type I error under control, we regard coefficients significant if p < .01, these coefficients are in bold

The analysis revealed that the variance of the general (composite) Somatic symptoms factor was significantly explained by the age, the diagnosis, and the perceived stress, of which age and perceived stress remained significant at the more restrictive significance level. The general (composite) Psychosocial symptoms factor was significantly explained by all four predictors, only the perceived stress reached the stricter criterion of significance. However, the association of perceived stress with the general Psychosocial symptoms factor was significantly stronger than with the general Somatic symptoms factor (Wald test = 24.7, p < .001).

Significant associations were also found for specific factors, even when controlling for general factors. Higher age predicted milder cognitive problems and anxiety and more severe eye symptoms. Gender differences were only found in one specific factor, but this did not reach the strictest significance level. In terms of diagnosis, those with GD reported milder cognitive problems, more severe hyperthyroid and eye symptoms, and cosmetic complaints. There was also a difference in the Impaired daily life factor in the diagnosis, but its significance level did not reach the predefined criterion. Perceived distress was significantly associated with anxiety, depressivity, and emotional susceptibility, even after controlling for general factors.

## Discussion

The Hungarian version of the ThyPRO-39 questionnaire is appropriate for measuring both general and specific factors of HRQoL in people with autoimmune thyroid disease. Previous research [23, 24] has mainly suggested the use of the composite score only for psychosocial symptoms, and our research supports this for somatic symptoms as well. However, the specific scores also carry information in addition to the composite scores and should be used when a more detailed analysis is required.

Our results supported that two general factors could be identified, suggesting the use of two composite scale scores to evaluate the HRQoL associated with autoimmune thyroid disease. On the one hand, the general factor of somatic symptoms may reflect the severity of the disease. On the other hand, the general factor of psychosocial symptoms may reflect its effect on well-being and psychosocial functioning. The two general factors correlate with each other; however, the nature of this correlation is not clear. It may reflect the impact of somatic symptoms on the psychosocial functioning, or it can be seen how psychosocial functioning has an impact on the disease symptom reporting. Longitudinal research would be needed to clarify the complex relationship between somatic symptoms and psychosocial functioning in this patient group.

Beside the general factors, most somatic symptom scales carry specific meaning. However, in the case of the Hypothyroid and Hyperthyroid symptoms scale the incremental explained variance is lower compared to the others. These scales measure symptoms that are not clearly thyroid-specific; they can connect to other health conditions or other characteristics, such as stress. Furthermore, many of the items of these scales can be connected to both hyper- and hypoactive thyroid functions [33, 34].

Interestingly, only two of the psychosocial-specific symptom scales — Cognitive problems and Emotional susceptibility — had remarkably high variance explained by specific meaning compared to the general factor. These results showed that valuable information could be gathered with the inclusion of these scales in the evaluation of patients’ psychosocial HRQoL.

Depending on the goals, both the composite and the specific scales can be used in the evaluation of HRQoL [23, 24]. The psychosocial scale can be helpful to measure the general impact of the disease/treatment on well-being and functioning and when comparing the burden of the different thyroid diseases. When a detailed evaluation is warranted, it is useful to add the specific well-being scales. Similarly, when we would like to quantify the specific somatic symptoms, we can use the separate, specific physical symptom scale. Still, when we aim to explore the frequency of physical symptoms or their changes in general, it is enough to use the Somatic symptoms (composite) scale.

The construct validity of the ThyPro-39 questionnaire was confirmed by the association with the level of perceived stress and general well-being. Both composite factors were significantly connected to these constructs. As we expected, the associations with the Psychosocial symptoms factor were significantly stronger than with the Somatic symptoms factor. The nature of the relationship between perceived stress and the two general factors is not yet clear. Is the higher perceived stress a consequence of the symptoms and psychosocial impact of the disease, or is it the stress that exacerbates these symptoms? We may assume that both mechanisms may be involved; however, further research should clarify.

The differences between the two autoimmune groups also confirmed the known-group validity of the ThyPro-39 questionnaire. GD patients reported more eye symptoms and cosmetic complaints. The autoimmune process in GD stimulates the TSH receptors, leading to unregulated thyroid hyperfunctioning in most cases [35]. GD may also be accompanied by orbital manifestations [8]; 25–50% of the patients develop Graves orbitopathy (GO), which explains the higher frequency of eye symptoms. GO severely affects HRQoL, primarily the appearance and social life [11, 36].

HT patients reported more severe hypothyroid symptoms and more cognitive problems. Hashimoto’s encephalopathy [37] may result in a decline in cognitive function, among other symptoms. Several mechanisms were described in the background: autoantibodies causing damage to nerve cells, depositing immune complexes destructing neurons through inflammation of blood vessels, and microthrombus processes. Hormonal pathways may also “support” the process [13, 38]; furthermore, hypothyroidism can affect the functioning of the hypothalamic-pituitary-adrenocortical (HPA) axis, which is also associated with cognitive and neuropsychiatric symptoms [38, 39]. Although GD patients also often suffer from neuropsychiatric symptoms, cognitive disturbances are not so prominent [11, 38]; instead, they appear mainly in the acute phase of Graves’ thyrotoxicosis [39].

Our result that older age predicts more somatic symptoms as longstanding autoimmune processes and thyroid failure seem to be associated with persistent, sometimes irreversible symptoms [9, 19, 37]. Presumably, it is not the age itself that matters, but the time since the onset of the disease that matters [18]. The negative association between age and cognitive and anxiety symptoms seems surprising but can originate from the low mean age of our sample. Getting diagnosed at a younger age can be more stressful and disturbing regarding cognitive functioning. Although the relationship between age and cognitive functions is not sufficiently explored [13].

One of the main limitations of our study is that we used self-reported diagnosis. Therefore, we could not control hormone levels and the activity of the underlying autoimmune processes. However, the biological variables are not necessarily crucial for exploring the factor structure and validity of the HRQoL questionnaire. Still, the use of clinical parameters would be advised in further studies when following HRQoL, its changes, and predictors.

## Conclusion

In conclusion, the Hungarian version of ThyPRO-39 is a valid and informative instrument and can be used for different purposes in clinical practice and research, such as the proper monitoring of the HRQoL among thyroid patients [12, 18], during treatments and the development of individualised care [10, 15, 23].

## Data Availability

The datasets used and analyzed during the current study are available from the corresponding author upon reasonable request.

## List of abbreviations

AITDs: Autoimmune thyroid disorders
CFA: Confirmatory factor analyses
CFI: Comparative Fit Index
ECV: Explained common variance index
GD: Graves’ disease
HPA: Hypothalamic-pituitary-adrenocortical
HRQoL: Health-related quality of life
HT: Hashimoto’s thyroiditis
MHCS-SF: Mental Health Continuum Short Form
PSS: Perceived Stress Scale
PU: Percentage of uncontaminated correlation
RMSEA: Root Mean Square Error of Approximation
ThyPro: Thyroid-Related Patient-Reported Outcome
